# Using a novel rapid alternating steering angles pulse sequence to evaluate the impact of theranostic ultrasound-mediated ultra-short pulse length on blood-brain barrier opening volume and closure, cavitation mapping, drug delivery feasibility, and safety

**DOI:** 10.7150/thno.76199

**Published:** 2023-02-05

**Authors:** Alec J. Batts, Robin Ji, Rebecca L. Noel, Alina R. Kline-Schoder, Sua Bae, Nancy Kwon, Elisa E. Konofagou

**Affiliations:** 1 Department of Biomedical Engineering, Columbia University, New York, USA; 2 Department of Radiology, Columbia University, New York, USA

**Keywords:** Focused ultrasound, cavitation mapping, phased array, short pulses, viral delivery

## Abstract

**Background:** Focused ultrasound (FUS)-mediated blood-brain barrier (BBB) opening is a noninvasive, safe and reversible technique for targeted drug delivery to the brain. Most preclinical systems developed to perform and monitor BBB opening are comprised of a separate geometrically focused transducer and passive cavitation detector (PCD) or imaging array. This study builds upon previous work from our group developing a single imaging phased array configuration for simultaneous BBB opening and monitoring called theranostic ultrasound (ThUS), leveraging ultra-short pulse lengths (USPLs) and a novel rapid alternating steering angles (RASTA) pulse sequence design for simultaneous bilateral sonications with target-specific USPL. The RASTA sequence was further employed to evaluate the impact of USPL on BBB opening volume, power cavitation imaging (PCI) pixel intensity, BBB closing timeline, drug delivery efficiency, and safety.

**Methods:** A P4-1 phased array transducer driven by a Verasonics Vantage ultrasound system was operated using a custom script to run the RASTA sequence which consisted of interleaved steered, focused transmits and passive imaging. Contrast-enhanced magnetic resonance imaging (MRI) confirmed initial opening volume and closure of the BBB by longitudinal imaging through 72 hours post-BBB opening. For drug delivery experiments, mice were systemically administered a 70 kDa fluorescent dextran or adeno-associated virus serotype 9 (AAV9) for fluorescence microscopy or enzyme-linked immunosorbent assay (ELISA) to evaluate ThUS-mediated molecular therapeutic delivery. Additional brain sections were also H&E-stained to evaluate histological damage, and IBA1- and GFAP-stained to elucidate the effects of ThUS-mediated BBB opening on stimulation of key cell types involved in the neuro-immune response, microglia and astrocytes.

**Results:** The ThUS RASTA sequence induced distinct BBB openings simultaneously in the same mouse where volume, PCI pixel intensity, level of dextran delivery, and AAV reporter transgene expression were correlated with brain hemisphere-specific USPL, consistent with statistically significant differences between 1.5, 5, and 10-cycle USPL groups. BBB closure after ThUS required 2-48 hours depending on USPL. The potential for acute damage and neuro-immune activation increased with USPL, but such observable damage was nearly reversed 96 hours post-ThUS.

**Conclusion:** ThUS is a versatile single-array technique which exhibits the potential for investigating a variety of non-invasive therapeutic delivery applications in the brain.

## Introduction

Many pharmacological treatment strategies for central nervous system (CNS) disorders are rendered ineffective primarily due to inhibited molecular transport of therapeutics in the brain. Passage of compounds greater than 400 Da in molecular weight from cerebral blood vessels into surrounding brain tissue is nearly entirely inhibited by existence of a specialized layer of endothelial cells called the blood-brain barrier (BBB) 1. Paracellular transport through the BBB is significantly hindered by tight-junction proteins which join these endothelial cells. The only existing non-surgical and targeted technique to bypass the BBB is focused ultrasound (FUS)-mediated BBB opening. FUS in conjunction with systemically administered acoustic contrast agents, called microbubbles, has been studied extensively over the past decade, and has been shown to be a safe and reversible technique for targeted drug delivery and modulation of the CNS 2-7 in both preclinical and clinical studies. Preclinically, BBB opening with FUS has been studied in the context of major diseases of the brain including brain tumors 8-10, or neurodegenerative disorders such as Alzheimer's disease (AD) 11,12 and Parkinson's disease (PD) 13-15. More recent clinical advances in FUS-mediated BBB opening demonstrated the potential for treating AD 16,17, amyotrophic lateral sclerosis (ALS) 18, and brain tumors 19. The primary underlying physical mechanism for FUS-mediated BBB opening is acoustic cavitation, the extent and nature of which can be modulated by altering FUS parameters or microbubble composition 20-23. Acoustic cavitation is primarily classified as either stable, characterized by rhythmic oscillations of microbubbles emitting subharmonic, harmonic or ultraharmonic frequency responses relative to the FUS center frequency 24, or inertial, which is historically associated with bubble implosion 24,25, increased potential for damage caused by increased FUS peak negative pressure (PNP) 21, and a broadband frequency response 26.

Given the association of acoustic cavitation and BBB opening, developing strategies to monitor cavitation activity during treatment has comprised another extensive body of research in the FUS community 27-30. Most FUS systems designed to both drive and monitor acoustic cavitation-mediated BBB disruption consist of a geometrically focused single element transducer with volumetric cavitation detection enabled by a separate single element passive cavitation detector (PCD), which, due to acoustic emission reception by only a single element, precludes spatial discrimination of cavitation signals 31-34. Alternatively, 2D cavitation mapping facilitated by a separate multielement imaging array 30,35 can provide advantageous spatial localization of cavitation activity unlike single-element PCD strategies. While most of these systems have been utilized to employ a long pulse regimen consisting of focused pulses on the order of 2,500-10,000 cycles to induce, record, and control acoustic cavitation activity, long FUS pulse lengths impede the success of high-resolution cavitation imaging techniques. Previous studies have demonstrated the utility of short pulse sequences on a microsecond scale, consisting of pulse lengths on the order of 1-10 cycles, to uniformly deliver up to 3 kDa-sized molecules across the BBB while accelerating BBB closure after disruption with FUS, posing both efficacy and safety advantages over long pulse sequences 36. Additionally, these short pulse regimens have permitted enhanced axial ultrasound imaging resolution and improved localization of microbubble activity 30.

While other investigators have designed and fabricated single transducer configurations for therapy and imaging 37-39, or have leveraged short pulses for accelerated BBB closure 36, our group has developed the only method repurposing a single diagnostic imaging probe for simultaneous BBB opening and high-resolution cavitation mapping with short therapy pulses, a novel technique called theranostic ultrasound (ThUS) 40. The study presented herein builds upon previous work from our group demonstrating the feasibility and safety of synchronous transcranial BBB opening and cavitation mapping through primate skull bone facilitated by electronic beam steering at a mechanical index (MI) of 0.4 40, to further capitalize on the technical flexibility afforded by a multielement imaging probe configuration. Specifically, we developed the novel rapid alternating steering angles (RASTA) pulse sequence for ThUS, leveraging electronic beam steering capability in conjunction with a research ultrasound computational interface to simultaneously induce and monitor BBB opening at multiple targets, each with distinct transmit parameter sets. While the aforementioned FUS configurations are capable of inducing BBB openings in the same subject within a single treatment session, most require physical translation of the transducer to each target, creating inconsistencies in cavitation activity between targets due to clearance of microbubbles from the vasculature between successive sonications when using a bolus injection paradigm 13,41, or achieve focal splitting via acoustic holograms at the expense of significant pressure loss through the lens material 42. Conversely, ThUS RASTA facilitated multi-spot BBB opening and target-specific high-resolution cavitation mapping in a single “shot”, with a single bolus injection of microbubbles. Furthermore, the RASTA pulse sequence permitted evaluation of, for the first time, the impact of ultra-short pulse lengths (USPL) on the order of 1.5-10 cycles on cavitation mapping pixel intensity, BBB closing timeline, molecular transport and viral delivery, acute microhemorrhage, and neuro-immune activation with multiple transmit parameter sets in the same animal. The results of this study emphasize both the potential for BBB opening with short pulses in particular applications such as cavitation guided-viral vector-mediated gene delivery, while postulating accelerated BBB repair using USPLs.

## Materials and methods

### Theranostic Ultrasound System

**Figure 1:**
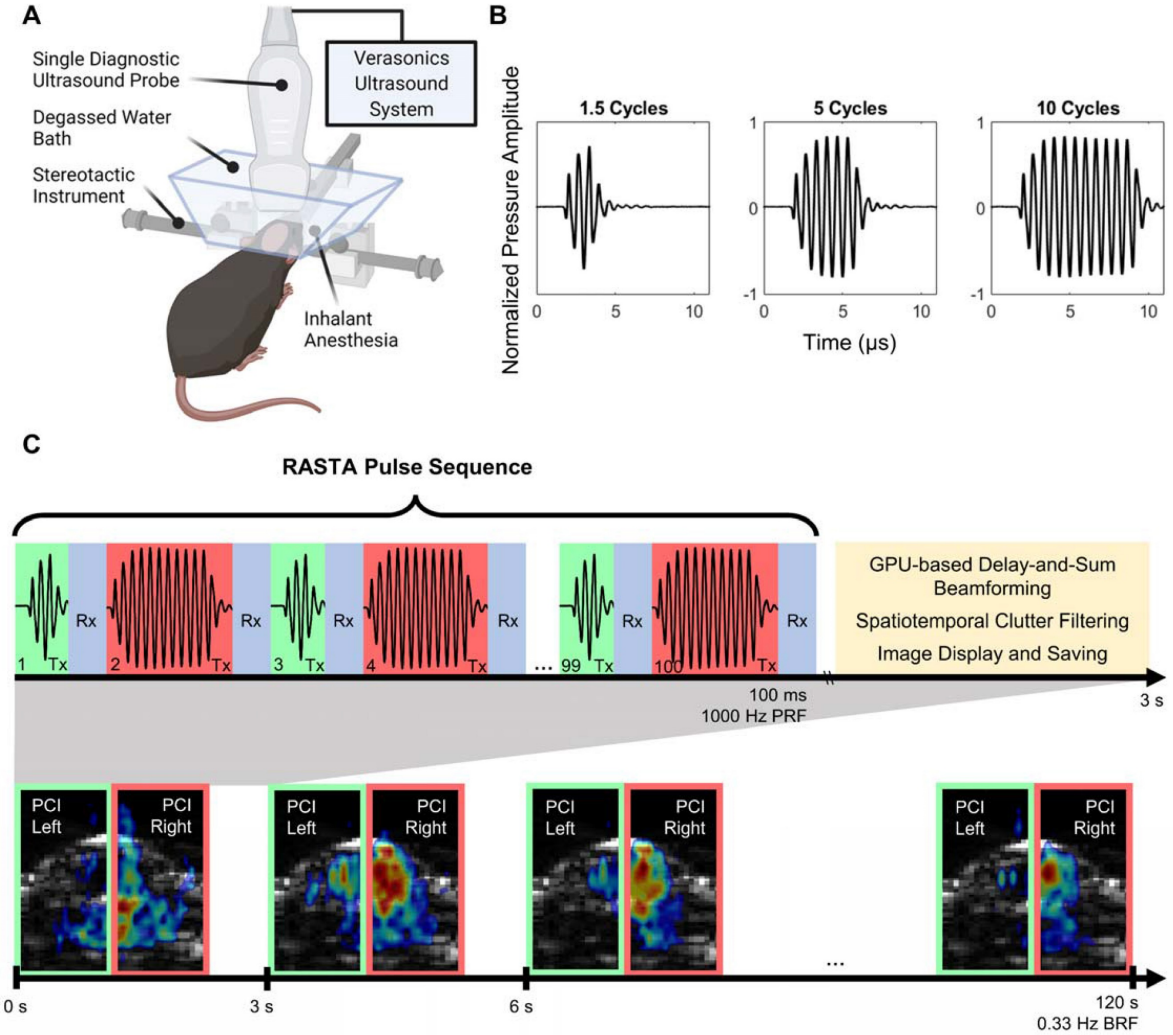
ThUS RASTA configuration and pulse sequence. **A)** Experimental apparatus for ThUS procedures. **B)** Experimentally recorded waveforms of the USPLs employed in this study. **C)** Overview of RASTA pulse sequence. Generation of each bilateral PCI is comprised of the RASTA transmit sequence, followed by GPU-based DAS beamforming, spatiotemporal clutter filtering and image display and saving. The RASTA sequence is comprised of alternating transmits of different USPLs (top panel): an example RASTA sequence deploying alternating 1.5-cycle transmits (shaded green) and 10-cycle transmits (shaded red) into the left and right hemisphere, respectively, separated by a brief period of passive receive mode (shaded blue). Bilateral PCI are generated in this manner every 3 seconds for the duration of the sonication (bottom panel).

A single transducer, multimodality configuration was employed for all BBB opening procedures in this study. A repurposed P4-1 (ATL, Philips) imaging phased array (2.5 MHz center frequency, 1.5-3.5 MHz -6 dB bandwidth, 96 elements) was operated by a research ultrasound system (Vantage 256, Verasonics Inc., Kirkland, WA, USA) at a frequency of 1.5 MHz (Figure 1A). A custom script was developed to combine power cavitation imaging (PCI) 30 and therapeutic focused transmits within the same transducer 40,43. The multi-element phased array design facilitated electronic focusing and steering of the beam with a -6 dB focal volume consisting of axial, lateral and elevational dimensions of 12.40 mm, 1.12 mm, and 7.43 mm (Figure S1A-B), which permitted development of flexible short-pulse sequence designs for simultaneous BBB opening and cavitation mapping. The RASTA pulse sequence was employed by defining interleaved transmit parameter sets, each with their own steering angle, using the Verasonics programming interface to electronically switch between these transmit parameter sets. The RASTA sequence developed in this study consisted of 100 total interleaved focused transmits emitted at a pulse repetition frequency of 1000 Hz, with odd numbered focused transmits deployed on the left hemisphere with a given USPL, and even numbered focused transmits deployed on the right hemisphere with a separate USPL (Figure 1C). Each transmit was separated by a period of passive receive mode for recording of cavitation activity induced by ThUS for PCI formation. The three USPLs evaluated in this study, 1.5, 5, and 10 cycles were input as half-cycles (3, 10, and 20 half-cycles) as required by the Verasonics sequence programming interface (Figure 1B). The RASTA sequence was immediately followed by a ~3 s period for GPU-based delay and sum beamforming, spatiotemporal clutter filtering, and PCI display and saving as previously reported 30,40. Each emittance of the RASTA sequence, repeated at a burst repetition rate (BRF) of 0.33 Hz, generated PCI for each hemisphere (Figure 1C) from which cavitation mapping pixel intensity was derived.

### Animals

All animal studies were approved by and performed in accordance with guidelines set forth by the Columbia University Institutional Animal Care and Use Committee (IACUC). Male C57BL/6J mice (Envigo, Indianapolis, IN, USA) were obtained at 4-6 weeks of age and housed for 2-4 weeks before ThUS experiments.

### Microbubbles

Polydisperse microbubbles for ThUS-mediated BBB opening procedures were manufactured in-house according to previously published protocols 40,44,45. In brief, 1,2-distearoyl-sn-glycero-3-phosphocholine (DSPC, Avanti Polar Lipids Inc., Alabaster, AL, USA) and 1,2-distearoyl-sn-glycero-3-phosphoethanolamine-N-[methoxy(polyethylene glycol)-2000] (DSPC-mPEG2000, Avanti Polar Lipids Inc., Alabaster, AL, USA) were dissolved at a 9:1 molar ratio in a glass vial containing a 50 mL solution of filtered phosphate-buffered saline (PBS) (40 mL), glycerol (10 mL), and propylene glycol (10 mL). The solution was submerged in a sonication bath maintained at 60 ℃ for 1-2 hours until all lipids were completely dissolved. The solution was allowed to cool before aliquoting into 5 mL vials for storage at 4 ℃ until use. Immediately before FUS procedures, air in the headspace of the vial was vacuumed out and replaced with perfluorobutane gas repeatedly for a total of 5 times, after which the vial was mixed and microbubbles were activated using a specialized shaker (VialMix^TM^, Lantheus Medical Imaging, N. Billerica, MA, USA) for 45 seconds. Resulting microbubble size distribution and concentration was evaluated using a particle sizer (Multisizer 4e Coulter Counter, Beckman Coulter, Indianapolis, IN, USA), where the calculated mean, median and mode sizes were 1.75 µm, 1.44 µm, and 1.01 µm, respectively. 5 µL of microbubbles thoroughly mixed in a 100 µL volume of either saline, diluted dextran, or AAV solution was injected into the tail vein immediately after initiation of the RASTA sequence, yielding a final microbubble concentration of 3.6×10^8^ MBs/mL.

### BBB opening procedure

Mice were anesthetized with a mixture of isoflurane and oxygen and maintained at a surgical plane of anesthesia for the duration of the BBB opening procedure. Anesthetized mice were restrained using a stereotaxic instrument (Model 900, David Kopf Instruments, Tujunga, CA, USA), and their heads were shaved and depilated to remove fur from the scalp. Ultrasound gel was degassed by centrifugation at 2000 rpm for 20 minutes at room temperature, and was applied over the scalp before placing a degassed water bath above the mouse head to facilitate acoustic coupling. Targeting of the focal region in the brain was achieved by first identifying the intersection of the lambdoid and sagittal sutures of the mouse skull, visualized through the skin of the depilated scalp, and placing a metal crosshair at lambda such that intersecting portions of the crosshair were oriented along the lateral and elevational dimensions of the transducer. The face of the P4-1 array was submerged in the water bath, and conventional B-mode imaging was used to center the ThUS beam in the elevational dimension by localizing the horizontal portion of the crosshair in the imaging field of view. Anatomical features and symmetry of the skull, visible on B-mode imaging, were then used to center the brain in the lateral dimension. The transducer was translated in the axial direction such that the center of the coronal brain slice at lambda intersected the point at a focal depth of 35 mm. The transducer was then translated 2.2 mm anterior from lambda for sonication. This B-mode targeting method permitted targeting with precision governed by the imaging resolution of the P4-1 phased array transducer operating at a frequency of 2.5 MHz. A B-mode image of the mouse head at the final targeted location was acquired for future registration of PCI. A tail vein catheter was inserted and saline was injected before applying the RASTA sequence to record and map baseline cavitation activity in the brain. The ThUS RASTA procedure was repeated with a bolus injection of microbubbles to induce BBB opening. For dextran or AAV delivery experiments, the microbubbles were co-injected with the compound at a concentration specified in the later respective sections. Approximately 40 bursts with corresponding PCI were acquired over a sonication duration of about 2 minutes. Two metrics of PCI pixel intensity were calculated over the sonication duration and used to correlate BBB opening volume with cavitation activity induced by ThUS: cumulative pixel intensity and peak pixel intensity. Cumulative PCI intensity was defined as the sum of the total pixel intensity over the duration of the ThUS sonication within an ROI corresponding to the imaging resolution afforded by each USPL (Figure S1C-E), whereas peak pixel intensity was defined as the maximum pixel intensity at the focal point using a fixed, 4-pixel ROI in order to reduce possible bias from using a resolution-adaptive ROI when determining cumulative PCI intensity. After the 2-minute sonication, the tail vein catheter was withdrawn and the mouse was removed from the stereotactic instrument and permitted to recover from anesthesia. Mice were sacrificed by transcardial perfusion with 4% paraformaldehyde (PFA) followed by PBS at a timepoint after the ThUS procedure according to the specific experimental requirements as specified later.

### Magnetic Resonance Imaging

MRI were acquired to confirm ThUS-mediated BBB opening, track BBB closure, and estimate permeability of the disrupted BBB with K_trans_. To confirm BBB opening, mice were intraperitoneally (IP) administered 0.2 mL of a gadolinium-based MR contrast agent (Omniscan, GE Healthcare, Princeton NJ, USA) and anesthetized with isoflurane throughout the scan in a 9.4T MRI system (Ascend, Bruker Medical, Billerca, MA, USA). T_1_-weighted 2D FLASH MRI (TR: 230 ms, TE: 3.3 ms, flip angle: 70°, 6 averages, FOV: 25.6 mm x 25.6 mm, matrix size: 256 x 256, slice thickness: 0.4 mm, resolution: 0.1 mm x 0.1 mm, scan time: 5 min) in axial and coronal planes were acquired 30 minutes after BBB disruption for initial confirmation of opening. To track BBB closure, a cohort of mice were administered another bolus injection of contrast agent before acquisition of the same T_1_-weighted MRI at 7 hours, 24 hours, 48 hours and 72 hours after BBB opening. To estimate closure on a finer timescale of 2 hours post-ThUS, several mice were given the same 0.2 mL bolus injection of contrast agent 1 hour and 30 minutes after ThUS to avoid confounding contrast enhancement signal with accumulation of remaining contrast agent if also previously injected immediately after ThUS. For estimation of permeability constant K_trans_, mice were anesthetized as previously described, catheterized IP, and inserted into the bore of the magnet before dynamic contrast enhanced (DCE) MRI imaging. A modified T_1_-weighted FLASH sequence (TR: 40 ms, TE: 1.4 ms, flip angle: 50°, 6 averages, FOV: 25.6 mm x 25.6 mm, matrix size: 160 x 160, slice thickness: 0.6 mm, repetitions: 55, scan time: 35 min) was initialized before injection of 0.3 mL contrast agent through the catheter during the 4^th^ repetition. The sequence of resulting images was used to estimate K_trans_.

### Pharmacokinetic modeling (K_trans_)

Images acquired from DCE-MRI were processed in a custom MATLAB script to estimate brain permeability to the MR contrast agent in the BBB opening regions using the general kinetic model 3,46. The transfer rate of contrast agent from blood to the extravascular compartment, referred to as K_trans_ was estimated in a 1.4 mm^3^ voxel of interest in the center of each BBB opening volume by comparing contrast agent accumulation to reference acquisitions before contrast agent administration.

### Histological preparation

Following the transcardial perfusion procedure described above, mouse brains were dissected, soaked in 4% PFA for 24 hours, followed by cryoprotection in 30% sucrose with 0.001% Sodium Azide for sectioning in-house, or preparation for paraffin embedding and hematoxylin and eosin (H&E) staining or ionized calcium binding adaptor molecule 1 (IBA1) and glial fibrillary acidic protein (GFAP) staining in 70% ethanol. For fluorescence imaging of delivered dextran and AAV, brains were coronally sectioned using a cryostat into 60 µm and 35 µm-thick slices, respectively, mounted on to slides, and coverslipped with 4',6-diamidino-2-phenylindole (DAPI) mounting solution (#ab104139, Abcam Inc., Waltham, MA, USA). For H&E, IBA1 and GFAP staining, brains were paraffin-embedded and coronally sectioned into 10 levels, with 4 consecutive sections per level of 5 µm-thick slices throughout the opening volume such that odd-numbered sections were stained for H&E, and even-numbered sections were stained for IBA1 and GFAP to associate neuro-immune activation with histological damage as follows. After deparaffinization, hydration, antigen retrieval and permeabilization, brain sections were incubated with 10% normal goat serum for 25 minutes at room temperature for blocking. Serum was removed and sections were incubated with IBA1 (1:200 dilution, #ab283346, Abcam Inc., Waltham, MA, USA) and GFAP antibodies (1:300 dilution, #ab7260, Abcam Inc., Waltham, MA, USA) overnight at 4 ℃. Sections were washed in PBS-T three times for 5 minutes each before addition of biotinylated goat anti-rat secondary antibody (1:200, #BA-9400-1.5, Vector Laboratories, Newark, CA, USA) and incubation for 30 minutes at room temperature. After washing, sections were incubated with Streptavidin, Alexa Fluor 488 conjugate (1:300, #S-11223, Invitrogen, Waltham, MA, USA), washed again, and incubated with goat anti-rabbit, Alexa Fluor 594 (1:400, #A-11037, Invitrogen, Waltham, MA, USA). Sections were coverslipped with mounting medium containing DAPI (#H-1500, Vector Laboratories, Newark, CA, USA) after a final round of washing.

### Dextran

70 kDa molecular weight dextran, fluorescently tagged with Texas Red (#D1830, Invitrogen, Waltham, MA, USA) was prepared according to the manufacturer's recommendation. A dose of 60 µg/g of mouse weight was co-injected with microbubbles via the tail vein catheter for ThUS-mediated dextran delivery.

### Adeno-associated Virus

The AAV9-CAG-GFP construct used in this study was developed by Edward Boyden and purchased from Addgene (Addgene viral prep #37825-AAV9) at a titer of 2.6×10^13^ gc/mL and stored at -80 ℃ until use. AAV was diluted to 1.3×10^12^ gc/mL, 6.5×10^11^ gc/mL and 1.0×10^11^ gc/mL and co-injected with microbubbles via the tail vein catheter at a total volume of 100 µL for ThUS-mediated viral delivery, corresponding to doses of 1.3×10^11^ gc/mouse, 6.5×10^10^ gc/mouse, and 1.0×10^10^ gc/mouse, respectively.

### ELISA

After sacrificing mice which received systemic administration of AAV four weeks prior, brains were dissected and split into individual hemispheres while peripheral organs were harvested and immediately frozen at -80 ℃ for transgenic protein quantification by enzyme-linked immunosorbent assay (ELISA). Tissues were thawed on ice before homogenization using either disposable micro tissue homogenizers for brain hemispheres (BioMasher, Takara Bio USA, Inc., San Jose, CA, USA) or a motorized tissue homogenizer with disposable pestles for peripheral organs. Tissues were homogenized in a volume of sterile 1x PBS normalized to the weight of tissue at a ratio of 1 g of tissue to 10 mL of PBS. After homogenization, samples were immediately processed by ELISA (GFP ELISA Kit, Fluorescent, #ab229403, Abcam Inc., Waltham, MA, USA) according to the manufacturer's instructions. Reported GFP concentrations represent picograms per milliliter of tissue homogenate.

### Microscopy

Fluorescence images of delivered dextran and viral delivery-mediated GFP transgene expression were acquired using 2.5x and 10x dry objectives (Leica DM6 B, Leica Microsystems Inc., Buffalo Grove, IL, USA). Bright-field images of H&E-stained sections were acquired from an automatic whole slide scanning system at 40x magnification (Leica SCN400, Leica Microsystems Inc., Buffalo Grove, IL, USA), and exported using the Aperio ImageScope software (Version 12.4.3, Leica Biosystems Imaging Inc., Buffalo Grove, IL, USA). Immunofluorescence images of IBA1 and GFAP stained brain sections were acquired using a 20x dry objective on a confocal microscope (LSM 700, Zeiss Microscopy, Jena, Germany).

### Image processing

Contrast enhancement on T_1_-weighted MRI was quantified using a custom MATLAB script to estimate BBB opening volume. For each axial slice, the area of contrast enhancement at a fixed threshold of two standard deviations above control region pixel intensity was calculated and summed across all slices, taking into account the MRI voxel size, to estimate the BBB opening volume. For fluorescence images, an analogous MATLAB script was used to quantify the area and optical density of fluorescent enhancement in each image after conversion to grayscale. Normalized optical density (NOD) metrics reported in the results section were calculated by dividing the average fluorescent intensity of pixels above a fixed threshold of three standard deviations over the average fluorescent intensity of a control region segmented from the unsonicated midbrain region.

### Statistical Analysis

All statistical analyses presented herein were performed using Prism (Version 9.20, GraphPad Software, San Diego, CA). Comparison of means between USPL groups were analyzed with one-way analysis of variance (ANOVA) with post-hoc Tukey's multiple comparisons test to determine statistical significance. Unless otherwise noted, statistical significance was classified with *p* < 0.05 and denoted by the following: *p < 0.05, **p < 0.01, ***p < 0.001, ****p < 0.0001. In all boxplots presented, whiskers represent the range of data. Numerical values reported within the text are represented as the mean ± standard error of the mean.

## Results

### ThUS RASTA simultaneously induces multiple distinct BBB openings by way of electronic beam steering and target-specific transmit parameter sets

**Figure 2:**
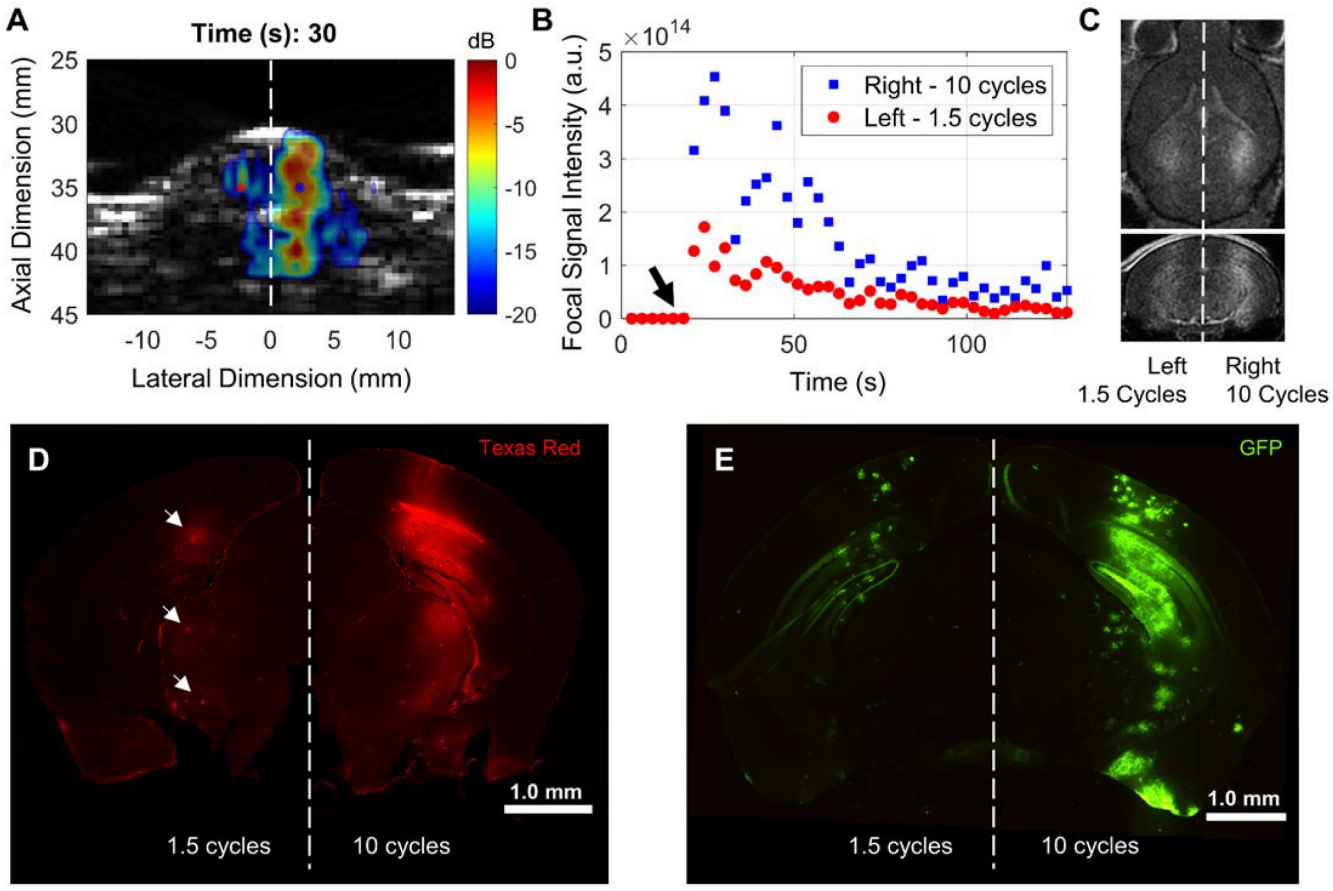
Differential BBB opening and drug delivery performance with ThUS RASTA. **A)** Representative display of PCI during sonication after microbubble injection in a mouse sonicated with the 10 cycle USPL on the right hemisphere and 1.5 cycle USPL on the left hemisphere, displaying qualitatively increased axial resolution given the shorter USPL on the left hemisphere. The center of the ThUS focus is denoted by the blue and red asterisks on the right and left hemispheres, respectively. **B)** Quantification of PCI pixel intensity at the focal point over the 2-minute sonication duration. The black arrow denotes the time at which the bolus injection of microbubbles was administered, while the blue squares and red points indicate focal PCI pixel intensity for the right and left hemispheres, respectively. **C)** Corresponding contrast-enhanced T_1_-weighted axial (top image) and coronal (bottom image) MRI acquired 30 minutes after sonication, displaying qualitatively increased contrast enhancement and BBB opening volume on the right hemisphere sonicated with the 10-cycle USPL relative to the left hemisphere. **D)** Whole coronal section of a mouse brain depicting ThUS-mediated delivery of 70 kDa Texas Red-tagged fluorescent dextran. Small pockets of dextran delivery were observed with reduced fluorescent intensity (white arrows). **E)** Whole coronal section of a mouse brain depicting ThUS-mediated delivery of AAV9-CAG-GFP at a dose of 1.3×10^11^ gc/mouse.

We configured the RASTA pulse sequence to sonicate multiple targets, based on anatomical symmetry, during the same sonication duration by interleaving steered focused transmits with corresponding cavitation imaging for each set of transmits. This flexibility enabled intra-subject investigation of effects of variations in USPL with fine granularity on PCI resolution, PCI pixel intensity, BBB opening volume and closing rate, in addition to delivery of several molecular therapeutic delivery agents. A demonstration of the beam steering-enabled ThUS RASTA sequence is shown in Figure 2. In addition to 1.5-fold increased focal pixel intensity in power cavitation images of the hemisphere targeted with the 10-cycle USPL relative to the 1.5-cycle USPL (Figure 2A-B), distinct BBB openings were induced, where the 10-cycle USPL elicited an increased BBB opening volume as confirmed by contrast-enhanced T_1_-weighted MRI (Figure 2C). Differential delivery performance of a 70 kDa dextran was also observed with clear increases in fluorescent intensity on the hemisphere targeted with the 10-cycle USPL relative to the 1.5-cycle USPL, which displayed small pockets of dextran accumulation throughout the focal volume (Figure 2D). Furthermore, distinct patterns of gene delivery were observed as an effect of USPL to deliver the GFP reporter transgene via AAV9 with the CAG promoter, where the 10-cycle USPL elicited visibly increased coverage of GFP expression throughout the entire depth of the mouse brain compared to the 1.5-cycle USPL, which exhibited low levels of transgene expression throughout regions of the focal volume towards the base of the brain (Figure 2E). The RASTA sequence permitted induction of distinct BBB openings supported by corresponding changes in pixel intensity of power cavitation images and drug delivery efficiency in multiple targets in the same animal, a sonication protocol which proved useful in further characterizing the effect of varying USPL.

With ThUS RASTA we investigated the impact of three USPLs, 1.5 cycles (1.0 μs), 5 cycles (3.33 μs), and 10 cycles (6.67 μs), on BBB opening volume and closing timeline, power cavitation imaging pixel intensity, drug delivery performance, and safety.

### BBB opening volume, permeability coefficient K_trans_, and power cavitation imaging pixel intensity increased with USPL

**Figure 3:**
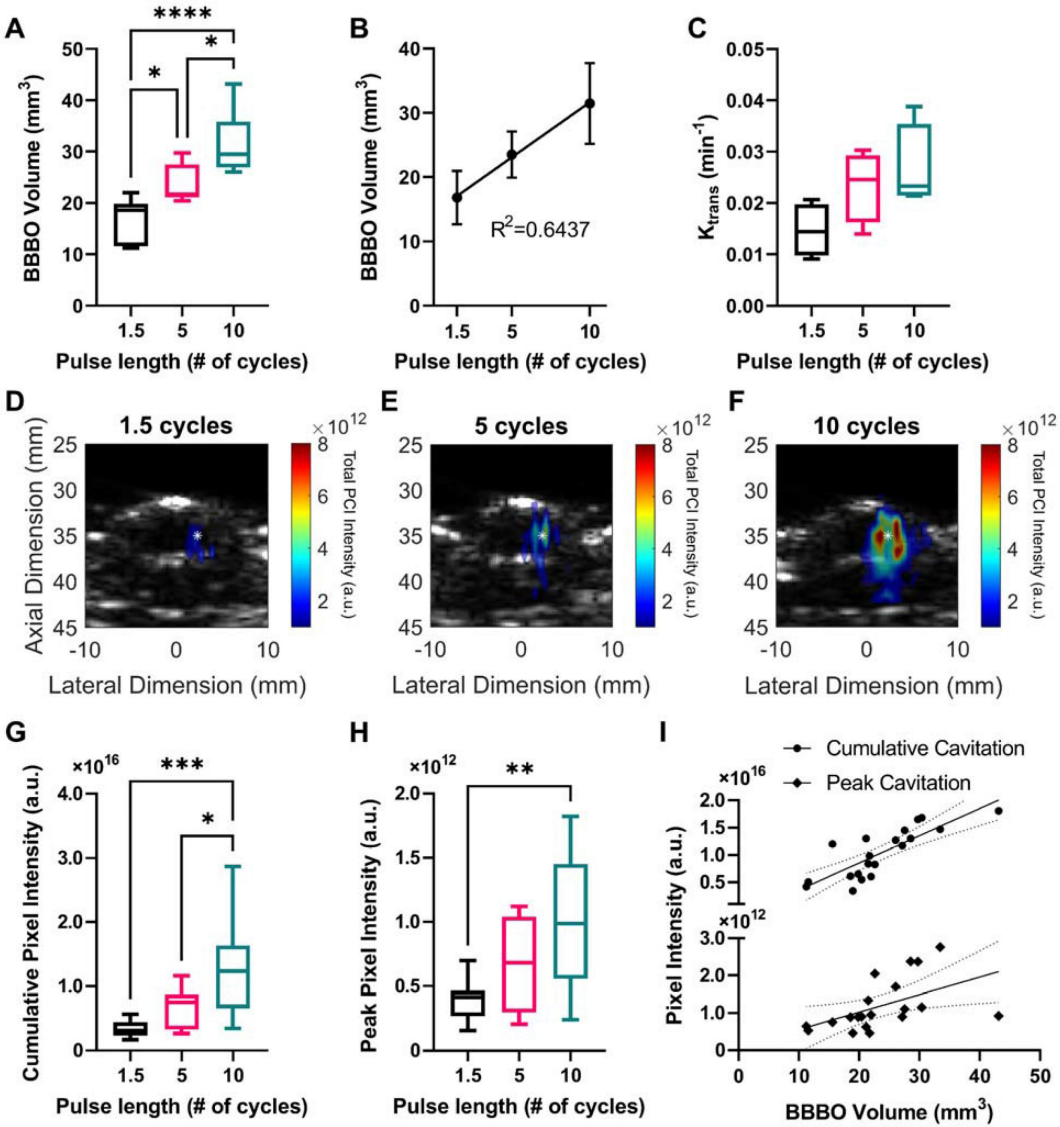
Effect of USPL on ThUS-mediated BBB opening volume, permeability and PCI pixel intensity.** A)** Statistically significant increases in BBB opening volume, quantified from contrast-enhanced T_1_-weighted MRI, with USPL were observed (*p < 0.05, ****p < 0.0001, n = 6-7 per group). **B)** Linear relationship between BBB opening volume and USPL (R^2^ = 0.6437). **C)** Trending increases in BBB permeability, K_trans_ with USPL. A significant linear trend in K_trans_ vs. USPL was detected (*p* < 0.05, slope = 0.0060 ± 0.0024). **D-F)** Representative PCI maps of total pixel intensity summed over the sonication duration for 1.5-cycle (D), 5-cycle (E), and 10-cycle (F) USPLs overlaid onto a pre-treatment cross-sectional B-mode image of the mouse skull and brain. White asterisk denotes center of the ThUS focus. Statistically significant increases in cumulative PCI pixel intensity **(G)** and peak pixel intensity **(H)** with USPL were observed (*p < 0.05, **p < 0.01, ***p < 0.001, n = 10-11 per group). **I)** Linear relationship between PCI pixel intensity and BBB opening volume (cumulative PCI pixel intensity vs. USPL: R^2^ = 0.6677, peak PCI pixel intensity vs. USPL: R^2^ = 0.2578). Solid black trendlines indicate line of best fit, while dashed lines indicate the 95% confidence interval. Statistical significance in (A), (G), & (H) determined by one-way ANOVA with post-hoc Tukey's multiple comparisons test. Coefficient of determination (R^2^) in (B), & (I) determined by standard linear regression. Linear trend in (C) confirmed by one-way ANOVA with post-hoc test for linear trend.

A cohort of mice was sonicated using ThUS RASTA and USPLs of 1.5, 5, and 10 cycles, where corresponding PCI were generated for each target. Contrast-enhanced T_1_-weighted MRI acquired immediately after the sonication confirmed significantly increased BBB opening volume with USPL as shown pictorially in Figure 2C, with an 87.09% increase in the average volume of opening induced by the 10-cycle USPL of 31.45 ± 2.57 mm^3^ relative to the average volume induced by the 1.5-cycle USPL of 16.81 ± 1.57 mm^3^ (Figure 3A). BBB opening volume was also linearly correlated with USPL with an R^2^ of 0.64 between BBB opening volume and USPL (Figure 3B). The effect of USPL on K_trans_ followed a similar trend with an 81.88% increase in K_trans_ from BBB openings induced by the 10-cycle USPL relative to the 1.5-cycle USPL (Figure 3C). While no statistically significant differences in mean K_trans_ were observed across USPL groups, a test for linear trend confirmed a significant linear relationship between K_trans_ and USPL. Representative PCI for each pulse length demonstrating an increase in total pixel intensity and qualitative decrease in axial resolution with USPL over the sonication duration, along with spatially accurate overlap with target coordinates in the brain, are shown in Figure 3D-F. Quantification of PCI cumulative and peak pixel intensities confirmed statistically significant increases in PCI pixel intensity with USPL, with up to a 3-fold increase in peak pixel intensity in the focal volume of the target sonicated with the 10-cycle USPL relative to the 1.5-cycle USPL (Figure 3G-H). Standard linear regression confirmed weak linear correlations between cumulative and peak pixel intensities, and USPL with R^2^ values of 0.4471 and 0.3239, while stronger linear correlations were observed between cumulative pixel intensity and BBB opening volume, with R^2^ = 0.6677 (Figure 3I), indicating that PCI may be a reliable ultrasound-based monitoring method for predicting BBB opening volume *in vivo*. Moreover, favorably resolved PCI enabled by ThUS and USPLs could be a promising improvement over the resolution of conventional passive acoustic mapping (PAM) techniques used in conjunction with long treatment pulse lengths.

### 70 kDa fluorescent dextran delivery increased with USPL

**Figure 4:**
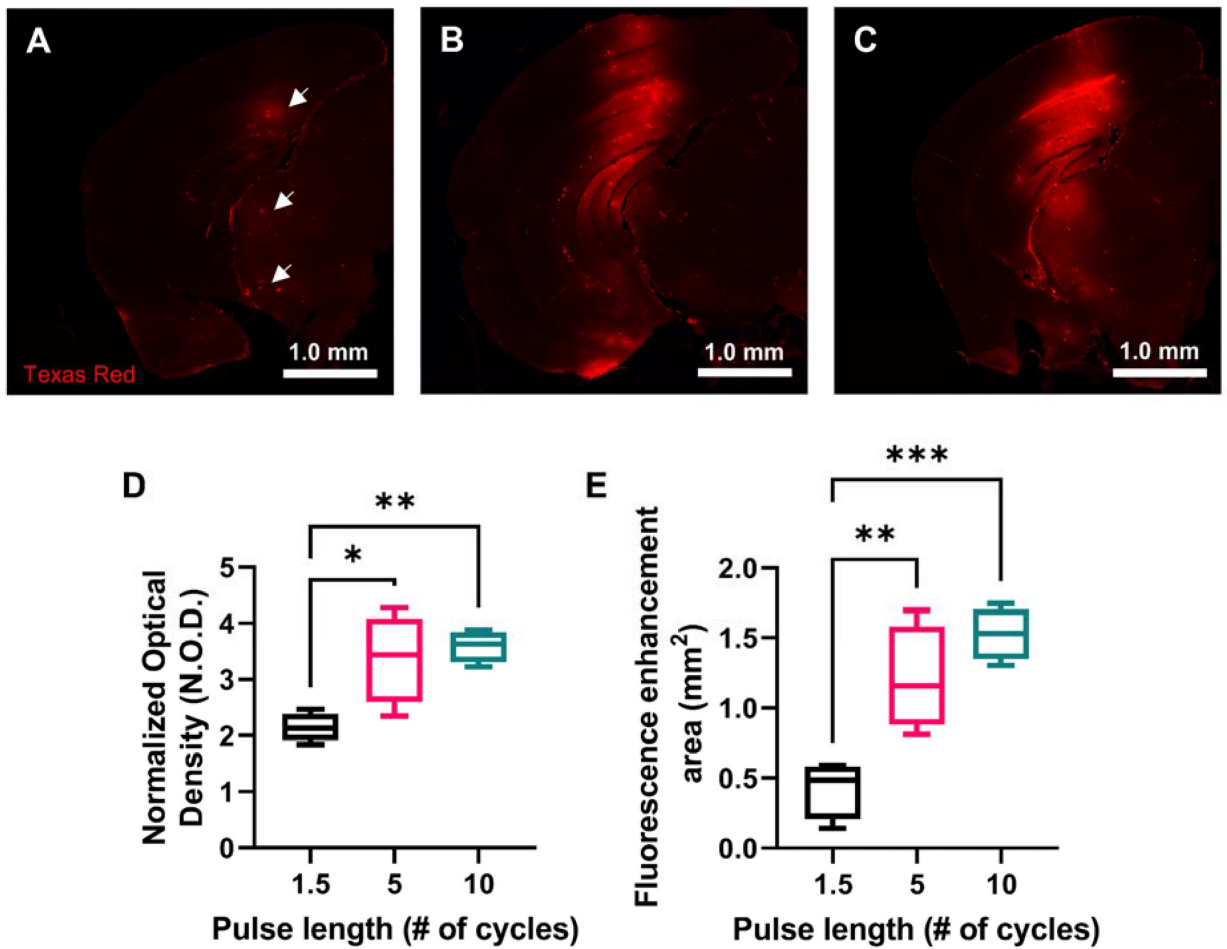
Effect of USPL on ThUS-mediated 70 kDa fluorescent dextran delivery. **A-C)** Whole hemisphere coronal sections of mouse brains sonicated with 1.5-cycle (A) 5-cycle (B) and 10-cycle (C) USPLs displaying increased fluorescent intensity within regions of BBB opening. White arrows in (A) depict small pockets of dextran extravasation facilitated by the 1.5-cycle USPL. **D)** Statistically significant increases in normalized optical density (NOD) with USPL. **E)** Statistically significant increases in area of fluorescent enhancement with USPL. Statistical significance in D-E determined by one-way ANOVA, followed by Tukey's multiple comparisons test. *p < 0.05, **p < 0.01, n = 4 per group.

In order to investigate drug delivery performance of each USPL evaluated using the ThUS RASTA sequence, a fluorescently-labeled 70 kDa Texas Red-tagged dextran was delivered bilaterally in another group of mice. Brains were harvested one hour after BBB disruption with ThUS, sectioned, and imaged to determine normalized optical density (NOD) and delivery coverage of the intravenously administered dextran. Microscope images of coronal brain sections at the center of the BBB opening region revealed increased dextran delivery with USPL (Figure 4A-C). Statistically significant NOD readouts of the fluorescently enhanced delivery regions were observed, particularly between the 1.5-cycle and 10-cycle USPLs (Figure 4D), where average NOD increased from 2.13 ± 0.13 in the 1.5-cycle group to 3.59 ± 0.14 in the 10-cycle group. Significantly increased delivery coverage indicated by the area of fluorescence enhancement in representative coronal brain sections at the center of the BBB opening volume was also observed as an effect of USPL (Figure 4E), with average delivery area ranging from 0.43 ± 0.10 mm^2^ in the 1.5-cycle group to 1.21 ± 0.19 mm^2^ and 1.53 ± 0.09 mm^2^ in the 5-cycle and 10-cycle groups, respectively. The similarity in NOD and delivery coverage between the 5-cycle USPL and 10-cycle USPL may also indicate an asymptotic trend for permeability of the BBB to 70 kDa-sized molecules in the context of BBB disruption with short pulses. Together, these results may also point to the potential for modulated delivery of neurotrophic factors or other proteins using ThUS given the similarity in molecular weight range.

### AAV9 delivery and GFP reporter gene expression increased with USPL

**Figure 5:**
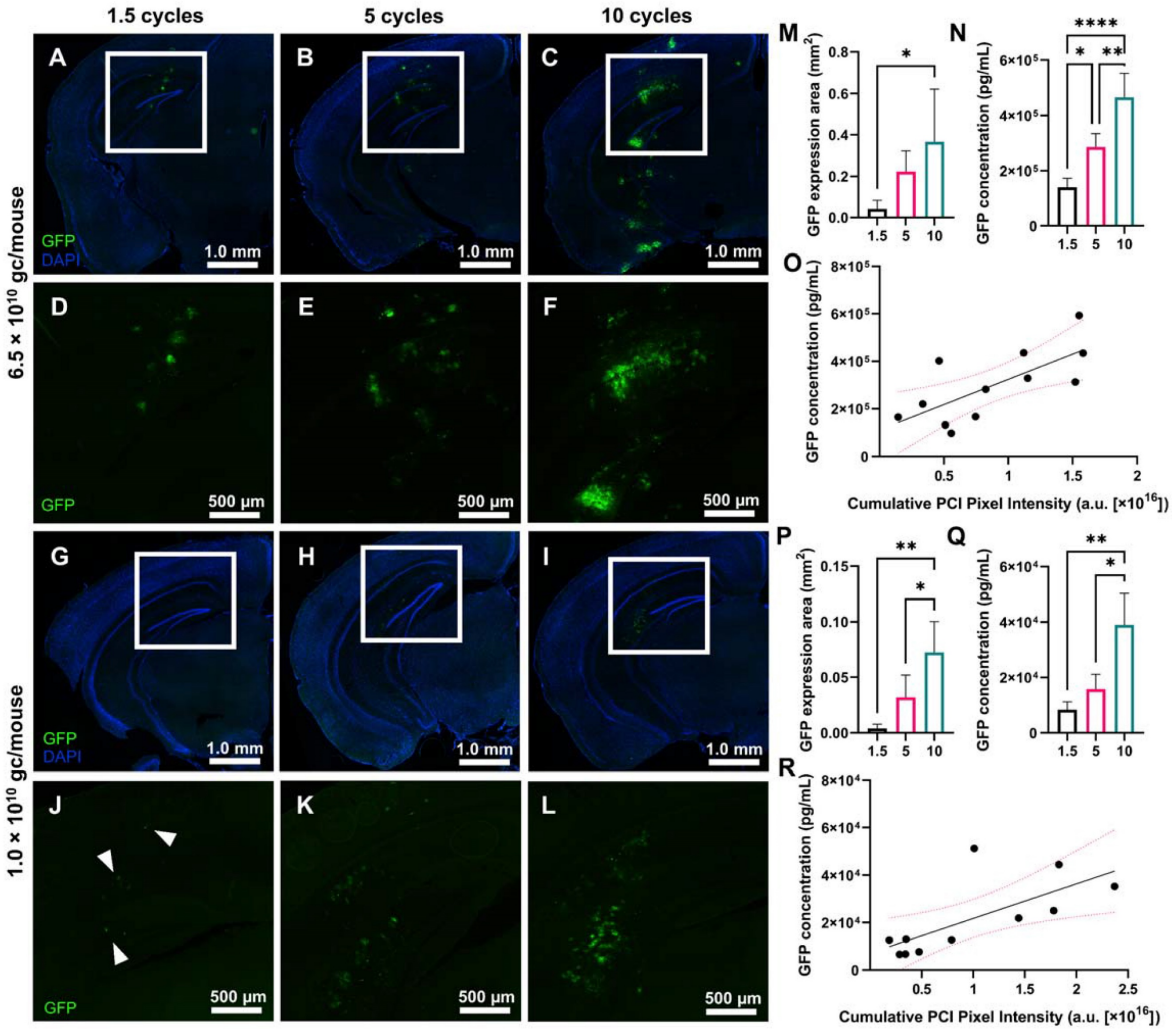
Effect of USPL on ThUS-mediated viral gene delivery performance. **A-C)** 2.5x magnified coronal sections of murine brain hemispheres harvested 4 weeks after ThUS-mediated AAV delivery at a dose of 6.5×10^10^ gc/mouse, sonicated with 1.5-cycle (A), 5-cycle (B), and 10-cycle (C) USPLs. DAPI is shown in blue, while GFP reporter transgene expression is shown in green. **D-F)** Corresponding 4x enlarged images of GFP transgene expression the cortical and hippocampal region outlined in the white rectangular ROI denoted in (A-C). **G-I)** 2.5x magnified coronal sections of murine brain hemispheres harvested 4 weeks after ThUS-mediated AAV delivery at a dose of 1.0×10^10^ gc/mouse, sonicated with 1.5-cycle (G), 5-cycle (H), and 10-cycle (I) USPLs. DAPI is shown in blue, while GFP reporter transgene expression is shown in green. **J-L)** 4x magnified images of analogous anatomical regions denoted by the white rectangular ROIs in (G-I), in mice which received AAVs at a dose of 1.0×10^10^ gc/mouse. GFP expression was observed in individual cells on hemispheres sonicated with 1.5 cycles, denoted by the white arrowheads in (J). **M)** Significant increases in GFP expression area observed with USPL at an AAV dose of 6.5×10^10^ gc/mouse. **N)** Significant differences in whole hemisphere GFP concentration quantified by ELISA at an AAV dose of 6.5×10^10^ gc/mouse. **O)** Linear correlation between cumulative PCI focal pixel intensity and whole hemisphere GFP concentration at an AAV dose of 6.5×10^10^ gc/mouse as determined by standard linear regression (R^2^ = 0.51, pink dashed lines indicate 95% confidence interval). **P)** Significant increases in GFP expression area observed with USPL at an AAV dose of 1.0×10^10^ gc/mouse. **Q)** Significant differences in whole hemisphere GFP concentration quantified by ELISA at an AAV dose of 1.0×10^10^ gc/mouse. **R)** Linear correlation between cumulative PCI focal pixel intensity and whole hemisphere GFP concentration at an AAV dose of 1.0×10^10^ gc/mouse as determined by standard linear regression R^2^ = 0.49, pink dashed lines indicate 95% confidence interval). Statistical significance in (M), (N), (P), (Q) determined by one-way ANOVA with post-hoc Tukey's multiple comparisons test. *p < 0.05, **p < 0.01, ***p < 0.001, ****p < 0.0001, n = 4 per group.

In addition to evaluating the ThUS-mediated delivery efficiency of molecules on the kilodalton scale, we also characterized the performance of larger viral vectors on the order of 4 MDa using ThUS, specifically uncovering the effect of USPL on GFP expression driven by AAV9 under the CAG promoter. AAV9-CAG-GFP was delivered using the ThUS RASTA sequence at two different doses, 6.5×10^10^ gc/mouse and 1.0×10^10^ gc/mouse, to uncover both the spatial distribution and whole brain concentration of transgenic protein expression while also determining the dose and USPL threshold for successful gene expression. After a survival period of 4 weeks after BBB opening with ThUS, mice brains were harvested, sectioned, and imaged for GFP expression or homogenized and processed for ELISA. Increasing USPL revealed increased GFP reporter gene expression in both dose groups (Figure 5A-L), with observable fluorescent enhancement indicating successful viral delivery in all mice sonicated with ThUS RASTA regardless of pulse length. For the 10-cycle and 5-cycle USPLs, gene expression was observed throughout the entire depth of the mouse brain at a dose of 6.5×10^10^ gc/mouse extending from the visual cortex through the CA1 region and dentate gyrus in the hippocampus, to the posteromedial and posterolateral cortical amygdaloid areas (Figure 5B-C, E-F), while gene expression was primarily observed in the visual cortex, CA1 region and dentate gyrus with the 1.5 cycle USPL (Figure 5A,D). In the lower dose group of 1.0×10^10^ gc/mouse, transgene expression was primarily localized to the CA1 hippocampal region and dentate gyrus (Figure 5G-L) across all USPLs evaluated, where the 1.5 cycle USPL elicited transgene expression in only a small number of cells within the BBB opening volume including pyramidal neurons in the CA1 hippocampal region through the visual cortex (Figure 5G,J).

Quantification of GFP fluorescent enhancement in coronally oriented brain slices selected from the center of the ThUS beam's elevational dimension shown in Figure 5 confirmed increasing GFP reporter transgene coverage with pulse length for both the 6.5×10^10^ gc/mouse dose group (Figure 5M) and 1.0×10^10^ gc/mouse dose group (Figure 5P). The average area of transgene expression increased from 0.042 ± 0.021 mm^2^ with 1.5 cycles, to 0.222 ± 0.051 mm^2^ with 5 cycles, and 0.367 ± 0.127 mm^3^ with 10 cycles at a dose of 6.5×10^10^ gc/mouse (Figure 5M) and 0.004 ± 0.002 mm^3^ with 1.5 cycles, to 0.032 ± 0.010 mm^3^ with 5 cycles and 0.072 ± 0.014 mm^3^ with 10 cycles at a dose of 1.0×10^10^ gc/mouse (Figure 5P). Whole-hemisphere GFP quantification by ELISA supported results of fluorescence microscopy for both AAV dose groups, where average GFP concentration at an initial dose of 6.5×10^10^ gc/mouse ranged from 1.41×10^5^ ± 1.62×10^4^ pg/mL with 1.5 cycles to 4.67×10^5^ ± 4.29×10^4^ pg/mL with 10 cycles (Figure 5N). At the reduced dose of 1.0×10^10^ gc/mouse, GFP concentration ranged from 8.41×10^3^ ± 1.42×10^3^ pg/mL with 1.5 cycles to 3.90×10^4^ ± 5.69×10^3^ pg/mL with 10 cycles (Figure 5Q), yielding an average fold-decrease in transgene concentration of 16.72, 18.03, and 11.96, for the 1.5 cycle, 5 cycle and 10 cycle USPL groups, respectively with a 6.5-fold decrease in dose (Figure S2A).

While input USPL significantly modulated transgene expression at both systemic dose groups, analysis of PCI acquired throughout the sonication was also conducted to evaluate the ability of PCI to predict viral vector delivery and transgene expression. A significant linear relationship between the cumulative PCI pixel intensity in the focal region and whole-hemisphere GFP concentration was observed in both the 6.5×10^10^ gc/mouse group (Figure 5O) and the 1.0×10^10^ gc/mouse group (Figure 5R), further demonstrating the utility of PCI as a reliable treatment monitoring tool for ThUS-mediated viral delivery.

In addition to quantifying brain transgene expression, peripheral organs were harvested and homogenized for evaluation of off-target systemic transgene expression with ELISA. Average GFP concentrations in the liver, kidney and heart at an initial dose of 6.5×10^10^ gc/mouse were 1.28×10^9^ ± 2.61×10^8^, 6.12×10^5^ ± 2.15×10^5^, and 1.44×10^5^ ± 5.24×10^4^, respectively, while a 6.5-fold reduction in systemic dose yielded an average fold-decrease in liver, kidney, and heart GFP expression of 2.67, 46.12 and 9.80, respectively (Figure S2B-D). Regardless of USPL used to deliver AAV in the brain, no significant differences in off-target transgene expression in the liver, heart, or kidney were observed across mice.

### Reducing USPL accelerated the rate of BBB closure

**Figure 6:**
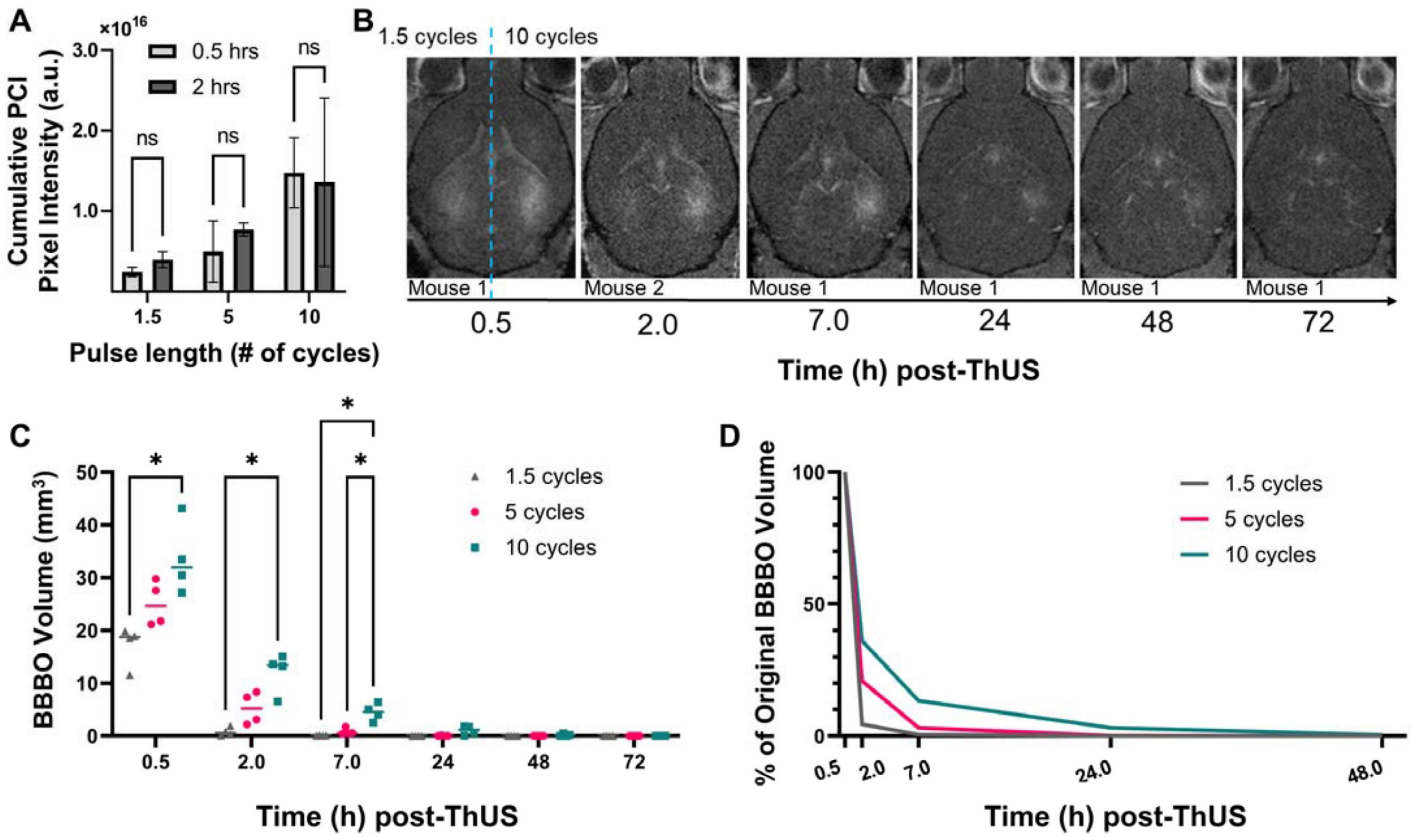
Effect of USPL on BBB closing timeline. **A)** Cumulative PCI pixel intensity quantification for mice which underwent MRI 0.5 hours post-ThUS versus 2 hours post-ThUS. Lack of statistically significant differences, determined by two-way ANOVA followed by Sidak's multiple comparisons test (n = 4 per group), in PCI pixel intensity indicates that initial BBB opening volumes between mice scanned at 0.5 hours and 2 hours were comparable. **B)** Temporal evolution of contrast enhancement in T_1_-weighted MRIs acquired serially after BBB disruption with ThUS. The right hemisphere of brains shown in (B) was sonicated with the 10-cycle USPL while the left hemisphere was sonicated with the 1.5 cycle USPL (hemispheres separated by blue dashed line). Note that images shown in (B) were serially acquired of the same mouse with the exception of the 2.0-hour image of another mouse brain given the temporal proximity of the two first timepoints and potential for contrast agent accumulation. **C)** Quantification of opening volume over time for each USPL. Statistically significant differences in opening volume between USPL groups were recapitulated at several timepoints, as determined by two-way ANOVA followed by Tukey's multiple comparisons test (*p < 0.05, n = 4 per group). **D)** Percentage reduction in BBB opening volume with time for each USPL, indicating a 95% reduction in contrast enhancement for the 1.5-cycle, 5-cycle and 10-cycle USPLs at 2.0 hours, 7.0 hours and 24 hours, respectively.

In addition to understanding the effects of USPL on initial opening volume and drug delivery performance, another cohort of mice was sonicated with the RASTA pulse sequence to determine differences in time required for BBB closure after disruption with ThUS. The volume of contrast enhancement on T_1_-weighted MRI was calculated for each target 0.5 hours, 2 hours, 7 hours, 24 hours, 48 hours and 72 hours after BBB disruption with ThUS, and each BBB opening was considered closed at the timepoint where a 95% reduction in initial opening volume was observed. Given the inability to administer contrast agent prior to MRI acquisition at 0.5 hours and 2 hours in the same mouse due to the close temporal proximity of these two timepoints and risk of accumulation of residual contrast agent, MRI at only the 2-hour timepoint were acquired on a separate cohort of mice. Comparison of cumulative pixel intensity from PCI acquired during ThUS RASTA from the cohort of mice scanned at 0.5 hours and the cohort of mice scanned at 2 hours revealed no statistically significant differences across cohorts (Figure 6A), indicating that initial BBB opening volumes were comparable across the two cohorts of mice.

As depicted qualitatively in the series of contrast-enhanced T_1_-weighted MRI shown in Figure 6B, the 10-cycle USPL group exhibited detectable contrast enhancement through 48 hours, with openings considered closed by 72 hours, while in the 1.5-cycle USPL group, a substantial decrease in contrast enhancement was observed by 2 hours, with openings considered closed by 7 hours. Though not presented in Figure 6B, openings induced by the 5-cycle USPL were considered closed by 24 hours under the same criteria (Figure 6C). A quantitative summary of these results is shown graphically in Figure 6C where average opening volumes fell below 3.0 mm^3^ by 24 hours for all USPLs evaluated. Additionally, statistically significant differences in opening volume were conserved through the 7-hour timepoint, further confirming the modulatory effect of USPL on BBB opening volume and reversibility. Clear stratification in the percent reduction in opening volume at each timepoint dependent on USPL was also detected (Figure 6D) demonstrating accelerated BBB closure as a potential benefit of BBB disruption with ThUS.

### Incidence of reversible histological damage and disruption of neuro-immune homeostasis increased with USPL

**Figure 7:**
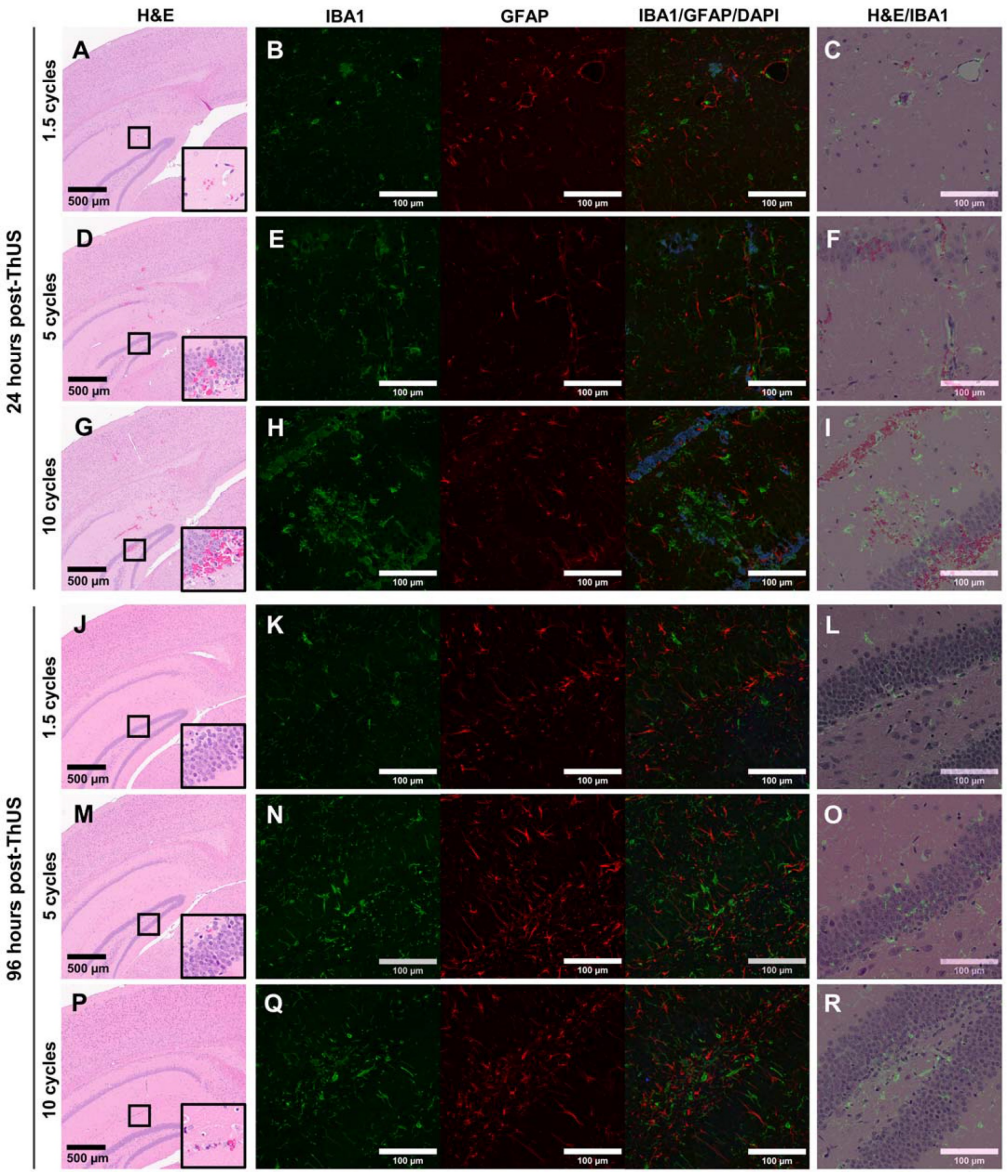
Effect of USPL on histological damage and neuro-immune activation. **A)** 4.6x magnified image of H&E-stained coronal brain section, **B)** 20x confocal microscopy images of IBA1 and GFAP staining for microglia and reactive astrocytes, and **C)** overlay of H&E and IBA1 images depicting aggregation of microglia surrounding regions of erythrocyte extravasation 24 hours post-ThUS exposure with the 1.5-cycle USPL. Corresponding images for the 5-cycle USPL 24 hours post-ThUS are shown in **D)** H&E, **E)** IBA1 and GFAP, and **F)** H&E and IBA1 overlay, and for the 10-cycle USPL 24 hours post-ThUS in **G)** H&E, **H)** IBA1 and GFAP, and **I)** H&E and IBA1 overlay. **J)** Image of H&E staining 96 hours post-ThUS for the 1.5-cycle USPL, along with **K)** IBA1 and GFAP staining, and **L)** H&E and IBA1 overlay. Corresponding images for the 5-cycle USPL 96 hours post-ThUS are shown in **M)** H&E, **N)** IBA1 and GFAP, and **O)** H&E and IBA1 overlay, and for the 10-cycle USPL 96 hours post-ThUS in **P)** H&E, **Q)** IBA1 and GFAP, and **R)** H&E and IBA1 overlay. Black squares in H&E images denote approximate location of inset in lower right corner. Inset width is 140 µm, black scale bars in H&E images are 500 µm, and white scale bars in immunofluorescence images and overlays are 100 µm.

In order to investigate potential histological damage and stimulation of two key neuro-immune cells, microglia and astrocytes, caused by ThUS at the MI and range of USPLs evaluated herein, two groups of mice were sacrificed either 24 hours, or 96 hours after BBB opening to detect acute microhemorrhages, track the temporal response of microglia and astrocytes to the applied USPLs, and assess the reversibility of microhemorrhage and disruption of neuro-immune homeostasis. At 24 hours post-ThUS, incidence of microhemorrhage within the hippocampus and cortex increased with USPL; the 1.5 cycle USPL elicited only minor erythrocyte extravasations (Figure 7A), while increased erythrocyte extravasation, particularly in the dentate gyrus was observed with the 5-cycle (Figure 7D) and 10-cycle (Figure 7G) USPLs. IBA1 staining revealed increasing aggregation of microglia with USPL (Figure 7B,E,H) which was interestingly associated with erythrocyte extravasations observed in manually registered adjacent H&E-stained sections (Figure 7C,F,I). Given that the peak in the astrocytic response is temporally delayed relative to that of microglia due to the chemokine and cytokine-mediated crosstalk between these two cell types 47, no apparent differences in GFAP density in reactive astrocytes were expected or observed across USPLs at the 24-hour timepoint.

96 hours post-ThUS, H&E staining revealed a dramatic reversibility in erythrocyte extravasations in all USPLs evaluated (Figure 7J,M,P). While no observable erythrocyte extravasations were observed in the 1.5-cycle USPL group, minor extravasations on the order of less than 10 cells were detected in the 5-cycle USPL (Figure 7M) and 10 cycle USPL (Figure 7P) groups. Additionally, IBA1 staining demonstrated a reduction in microglia aggregates, with only a small number of representative areas depicting clusters of morphologically-indicative non-homeostatic microglia in the 5-cycle USPL (Figure 7N) and 10-cycle USPL (Figure 7Q) groups. Given the aforementioned temporal delay in astrocytic activation following microglial responses, observable increases in GFAP expression by reactive astrocytes were expected and observed at the 96-hour timepoint, and were most evident in the 5-cycle (Figure 7N) and 10-cycle (Figure 7Q) USPL groups. Finally, overlaid H&E and IBA1 images at the 96-hour timepoint revealed that remaining regions of clustered microglia were no longer associated with erythrocyte extravasations at 96 hours relative to the 24-hour timepoint, implicating the important role of microglia-mediated erythrocyte phagocytosis 48,49 in restoration of the disrupted BBB after opening with ThUS.

## Discussion

This study constitutes the first investigation into the effect of USPL with ultra-fine granularity on efficiency of targeted therapeutic delivery to the brain in a novel and flexible imaging array platform. Leveraging the RASTA pulse sequence, we demonstrated target-specific BBB opening performance in the same animal, yielding differential BBB opening volume and noninvasive drug delivery capability corroborated by microbubble acoustic emission mapping from PCI. Even with ~2-3 μs increases in USPL, BBB opening volume and PCI pixel intensity were significantly modulated. As supported by previous studies associating pulse length with the BBB opening volume and K_trans_ 3, USPL was also found to be linearly correlated with both opening volume and K_trans_ in this study. Furthermore, increases in USPL also elicited significantly improved drug and viral vector-mediated gene delivery. Delivery of a 70 kDa fluorescent dextran with ThUS RASTA revealed both increasing fluorescence intensity and spatial coverage with USPL. This relationship was recapitulated in the context of viral delivery where gene transduction after AAV delivery with ThUS RASTA at two different systemic doses of AAV9 administration increased with USPL. Another previously elucidated benefit of BBB opening with short pulses, accelerated BBB closing timeline 36,40, was reiterated in the context of USPLs where the time required for restoration of impermeability of the BBB to a 591.7 Da gadolinium-based MR contrast agent was significantly reduced with a decrease in USPL. Finally, while increasing USPL exhibited drug delivery advantages, it also raised the probability for incidence of short-term histological damage and neuro-immune stimulation. However, damage induced by BBB opening with longer USPLs evaluated in this study was almost entirely reversed within 96 hours, indicating that the nature of the tradeoff between increased drug and gene delivery is characterized by acute, but not permanent microhemorrhage.

Given the technical limitations imposed by a dual-mode therapeutic and cavitation imaging technique conducted using a single diagnostic imaging phased array, transmit parameters were selected to both maximize drug delivery potential for a relatively large size range of compounds and preserve the advantage of high-resolution cavitation mapping. In this study, a 1.0 MPa *in situ* PNP was used in order to induce primarily broadband acoustic emissions from microbubbles and mitigate both pulse length and transducer bandwidth related constraints for cavitation mapping. Given that recording of stable harmonic acoustic emissions was precluded by the use of short pulses along with the relatively narrow bandwidth of the P4-1 phased array (1.5 MHz - 3.5 MHz), reception and mapping of broadband signal comprised the basis of the cavitation imaging method presented herein. Additionally, the choice of a fixed 1.0 MPa PNP allowed for evaluation of BBB opening and drug delivery performance of a selection of compounds with a broad size range by directly modulating USPL. This PNP was sufficient enough to induce variable transport of compounds ranging from 0.591 kDa gadodiamide MR contrast agent, to a 4 MDa AAV by maintaining a fixed pressure and altering USPL only. Furthermore, employing USPLs from 1.5-10 cycles minimized degradation of axial resolution inherent to cavitation mapping techniques used in conjunction with long-pulse transmit sequences.

Notable advantages of ThUS, a short-pulse regimen, and pulse sequence such as RASTA were revealed in this study. First, the rapidly accelerated closing timeline observed after BBB opening with 1.5-cycle USPLs, evident by a 95% reduction in MR contrast enhancement after just two hours, and contrast enhancement below the level of detection at 7 hours post-BBB opening, may be suitable for applications requiring highly precise gene transduction in only a small number of cells, or modulation of the CNS without inducing significant acute histological damage. While FUS parameters, animal models or patient populations, and methods for assessing BBB closure vary extensively across studies, making it challenging to evaluate the BBB closing phenomenon in the context of other literature in the BBB opening space, most preclinical studies conducted in rodents, employing non-damaging parameters, report BBB closure within 24 hours. Though the volume of BBB opening was not stated, Marty et al. (2012) reported BBB closure detected via lack of Dotarem (753.9 Da) contrast-enhancement within 24 hours in Sprague Dawley rats using 1.5 MHz FUS with an *in situ* PNP of 450 kPa and 3 ms-long pulse length 50. With a comparable FUS center frequency and PNP, yet more than a two-fold increase in pulse length, Samiotaki et al. (2013) reported BBB permeabilization surpassing 48 hours for a 15-25 mm^3^ initial opening volume 3, while with ThUS RASTA, openings of the same range of initial volume were closed by 7 hours. Morse et al. (2019) also observed absence of fluorescence enhancement from a 3 kDa dextran using a similar short-pulse regimen as presented herein when injected merely 10 minutes post-BBB opening, whereas fluorescent signal from the same dextran formulation is detected when injected 20 minutes post-BBB opening using a long-pulse sequence 36. Another elucidated advantage of the RASTA pulse sequence, enabled by the electronic beam steering flexibility of the P4-1 phased array, relates to the induction of bilateral BBB openings simultaneously after a single bolus injection of microbubbles; this eliminated any confounding variation across BBB openings caused by exposure of the ThUS beam to different concentrations of microbubbles in the cerebrovasculature, which is inevitable in a serial bilateral sonication paradigm using microbubble bolus injections. In such a scenario, microbubble clearance behavior between temporally-separated sonications may introduce inconsistent cavitation activity between targets. While specialized acoustic lensing techniques have been developed to enable simultaneous bilateral BBB opening 42, propagation of MHz-order ultrasound frequencies through the lens material elicits significant pressure attenuation, which, in a transcranial context where pressure attenuation due to the skull already limits BBB opening efficiency, may be overcome instead with alternative electronic acoustic lensing techniques such as the RASTA sequence.

Regarding the targeted delivery performance of ThUS achieved with RASTA, successful delivery of both 70 kDa dextran and AAV9, indicated by GFP transduction, demonstrates the potential for both protein and viral gene delivery in the context of disease. The plateau in fluorescent dextran NOD and delivery coverage observed with increasing USPL beyond 5 cycles indicates that for comparably sized compounds such as neurotrophic factors, increasing USPL increases potential for temporary damage without a substantial increase in delivery efficiency. Conversely, increasing USPL in both AAV dose groups yielded significant increases in GFP transduction. In all cases, pyramidal neurons of the hippocampus were transduced, indicating possible applications for ThUS-mediated viral delivery for improvements in memory, sensory or motor function. An interesting delivery pattern worth noting was also observed, particularly in the 10-cycle USPL group at an AAV dose of 6.5×10^10^ gc/mouse (Figure 5C). Gene transduction did not occur uniformly along the path of the ThUS beam extending from the visual cortex to the posteromedial cortical amygdaloid area, but rather appeared as splotches, which is a delivery pattern previously associated with inertial cavitation activity 33. However, the ability to noninvasively deliver therapeutics to deep structures within the brain with longer USPLs may allow access to structures which are challenging to reach with direct intraparenchymal injections. While the AAV dosing in this study was chosen to establish feasibility for gene transduction in the brain with a reduced systemic dose relative to previous viral delivery studies with FUS 13,51,52, a more uniform viral delivery pattern may require an increase in systemic dose given the nature of cavitation activity induced with USPLs.

While a number of advantages exist using a single phased array configuration in combination with USPLs for both bilateral sonication flexibility and imaging resolution, some limitations of our study necessitate discussion for further improvement. Given that the vast majority of BBB opening studies are conducted with long-pulse sequence designs, evaluating short-pulse treatment schema in the context of the larger FUS-mediated BBB opening literature space is challenging. For example, in previous studies from our group utilizing 6.7 ms-long pulses, a derated PNP of 0.45 MPa, and a 1.5 MHz transmit frequency, the corresponding mechanical index (MI), is approximately 0.36. In this study, usage of 1.0-6.67 μs pulses, a derated PNP of 1.0 MPa, and an analogous transmit center frequency of 1.5 MHz yields an MI of approximately 0.82. Given that MI greater than 0.5 are historically associated with incidence of damage via inertial cavitation 53, though only minor damage was observed after 1.5-cycle transmits in this study, the inference of BBB opening safety from MI in a short-pulse sequence regimen is a challenging comparison to make with existing studies employing long pulses 54. Given the nature of the neuro-immune response following ThUS-mediated BBB opening at the MI of 0.82 chosen in this study, evaluation of reduced MI and further optimization of ThUS pulse sequences to maximize drug delivery while reducing the likelihood for acute microhemorrhage and subsequent microglial and astrocytic response is ongoing. Another limitation of the ThUS technique presented herein is that the association of BBB opening volume with stable harmonic and ultraharmonic cavitation signals detected through stable oscillatory engagement of microbubbles exposed to long pulses is precluded by the use of short pulses, where the PCI signal employed in this study represents primarily broadband acoustic emissions. This inconsistency in cavitation dynamics also contributes to the complexity of evaluating BBB opening performance induced by short pulses in the context of a large body of FUS-mediated BBB opening research performed with long pulse sequences. While ongoing work is being conducted to further understand the nature of cavitation activity induced by ThUS, for the purposes of this study, quantitative comparisons between PCI pixel intensity are relative and cannot be directly compared with cavitation doses reported in the literature for long-pulse BBB opening regimens. Additional ongoing work includes extensive transcriptomic analysis of neuro-immune stimulation following FUS- and ThUS-mediated BBB opening in order to provide a more comprehensive understanding of microglial and astrocytic roles in BBB repair apart from conclusions drawn from histopathological analysis alone.

Along with further understanding of cavitation activity induced by ThUS, additional ongoing work and future directions include further leveraging the flexibility of RASTA beyond alteration of USPL to also vary the transmit frequency between targets in the heterogeneous primate skull for optimal transcranial acoustic wave propagation. Furthermore, investigations aimed at uncovering the mechanism for BBB opening with short pulses are an immediate priority as our group continues to develop ThUS technology.

## Conclusion

In this study, we presented the novel RASTA pulse sequence for simultaneous BBB opening and monitoring in distinct coordinates in the murine brain using ThUS. Given the consistent bilateral BBB opening and drug delivery performance enabled by our single-transducer technique, further optimization and adaptation of ThUS technology for large animal models and humans may eventually present a low-cost, portable, fully ultrasound-guided strategy for drug and gene delivery in the clinic.

## Supplementary Material

Supplementary figures.

## Abbreviations

AAV: adeno-associated virus
AD: Alzheimer's disease
ALS: amyotrophic lateral sclerosis
ANOVA: analysis of variance
BBB: blood-brain barrier
BRF: burst repetition frequency
CNS: central nervous system
DCE: dynamic contrast enhanced
FUS: focused ultrasound
GFAP: glial fibrillary acidic protein
GFP: green fluorescent protein
ELISA: enzyme-linked immunosorbent assay
H&E: hematoxylin and eosin
IBA1: Ionized calcium binding adaptor molecule 1
IP: intraperitoneal
MI: mechanical index
MRI: magnetic resonance imaging
NOD: normalized optical density
PAM: passive acoustic mapping
PBS: phosphate-buffered saline
PCD: passive cavitation detector
PCI: power cavitation imaging
PD: Parkinson's disease
PFA: paraformaldehyde
PNP: peak-negative pressure
RASTA: rapid alternating steering angles
ThUS: theranostic ultrasound
USPL: ultra-short pulse length
