# Supplementary material for: Using a novel rapid alternating steering angles pulse sequence to evaluate the impact of theranostic ultrasound-mediated ultra-short pulse length on blood-brain barrier opening volume and closure, cavitation mapping, drug delivery feasibility, and safety
Source: Theranostics. 2023 Feb 5;13(3):1180–97. doi: 10.7150/thno.76199 (PMC9925313; doi:10.7150/thno.76199)
Supplement: Supplementary file 1 — Supplementary figures. [file thnov13p1180s1.pdf]

Supplementary Material

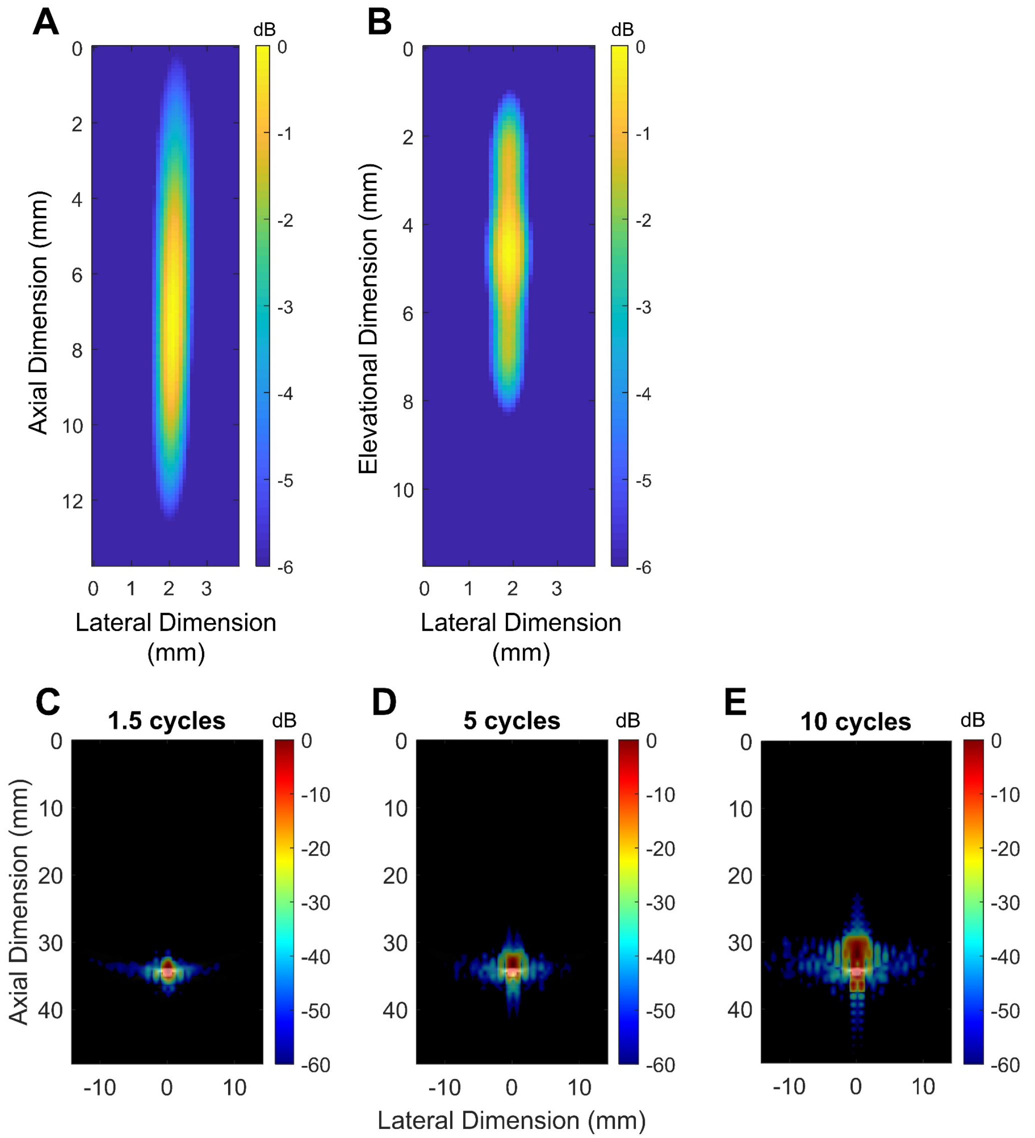

Figure S1: ThUS focal dimensions and PCI point spread function. **A)** -6 dB (pressure) focal region in axial/lateral plane. **B)** -6 dB focal region in elevational/lateral plane. PCI PSF overlaid onto B-mode image of a 0.30 mm-thick metal wire cross-section for **C)** 1.5-cycle, **D)** 5-cycle, and **E)** 10-cycle USPLs.

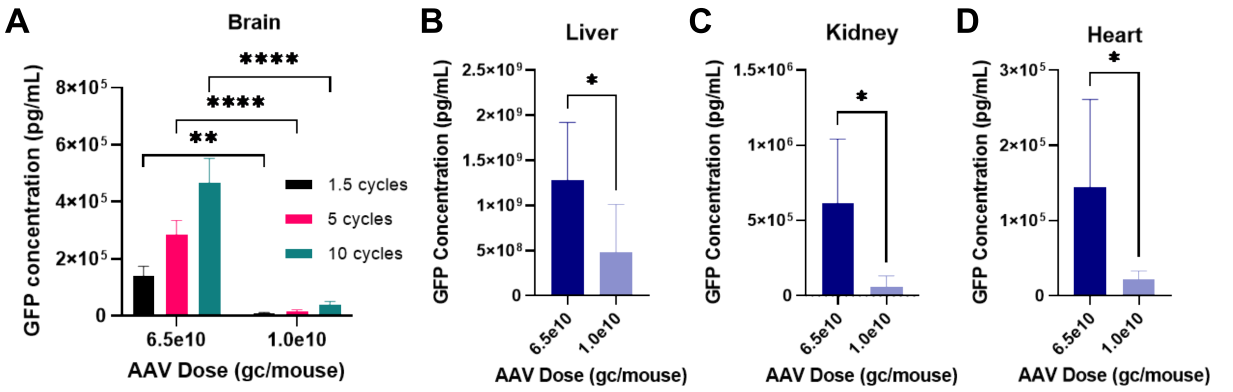

Figure S2: Reduction in brain and peripheral organ transgene expression with reduced systemic AAV dose. **A)** Significant decreases in whole brain hemisphere GFP concentration quantified by ELISA with AAV dose reduction at all USPLs evaluated. Significant decreases in **B)** liver, **C)** kidney, and **D)** heart GFP concentration with AAV dose reduction. Statistical significance in (A) determined by two-way ANOVA with Sidak multiple comparisons correction, and (B) by student's t-test. $^*p < 0.05$, $^{**}p < 0.01$, $^{****}p < 0.0001$, n = 4-6 per group.

4
